# Engaging Older Adults With Cognitive Impairment in Digital Health Technologies: Protocol for a Scoping Review

**DOI:** 10.2196/65515

**Published:** 2025-06-03

**Authors:** Sié Mathieu Aymar Romaric Da, Maxime Sasseville, Marie-Soleil Hardy, Idrissa Beogo, Amédé Gogovor, Samira Amil, Achille R Yameogo, Frédéric Bergeron, Anik Giguere, Annie LeBlanc, James Plaisimond, Carole Rivard-Lacroix, Marie-Pierre Gagnon

**Affiliations:** 1 Faculty of Nursing Sciences Laval University Quebec, QC Canada; 2 Library Advisory Services Department Laval University Quebec, QC Canada; 3 School of Nursing Ottawa University Ottawa, ON Canada; 4 Faculty of Medicine Laval University Quebec, QC Canada; 5 Institute for Aging and Social Participation of Seniors Laval University Quebec, QC Canada

**Keywords:** engagement, older adults, cognitive disorders, digital health technologies, scoping review

## Abstract

**Background:**

The aging of the population is a major issue in Canada, particularly in Quebec. For people older than the age of 65 years, aging is often associated with both mild and severe cognitive impairment. The management of these disorders increases the pressure on health care systems. Digital health technologies could be used to promote the cognitive health of older people living with cognitive disorders. However, to reap the full benefits of using digital health technologies, it is critical that older people with cognitive disorders engage with these technologies. A dose-response relationship has been demonstrated between the level of engagement with digital health technologies and the effectiveness of interventions in older people. It is thus important to understand how older people with cognitive impairment engage with digital health technologies and how this engagement can influence the success of digital health interventions.

**Objective:**

This study aims to describe how the engagement of older adults with cognitive impairment with digital health technologies is conceptualized and assessed, and how this engagement relates to the effectiveness of digital health interventions.

**Methods:**

We will use the scoping review method outlined by Arksey and O’Malley. We will apply a systematic approach following the Joanna Briggs Institute guidelines and the PRISMA-ScR (Preferred Reporting Items for Systematic Reviews and Meta-Analyses Extension for Scoping Reviews) checklist to ensure reproducibility of the scoping review. A search strategy, created with a medical information specialist, will be applied to MEDLINE, Embase, CINAHL, Web of Science, and Google Scholar, without time restrictions. Two reviewers will independently select titles, abstracts, and full texts. Data extraction will be conducted by the research team and validated by a senior member, resolving disagreements by consensus or a third party if necessary. Descriptive analyses will be done using concept mapping for a narrative synthesis of the results by themes related to the research questions.

**Results:**

The development of the search strategy and the completion of the selection phases of the review were completed in July 2024. Data extraction and analysis began in August 2024, and results are expected to be available in November 2024.

**Conclusions:**

The results of this scoping review will provide a comprehensive overview of the different conceptualizations of engagement with digital health technologies in older people with cognitive impairment, as well as the tools to measure it. This will contribute to a better understanding of the relationships between levels of engagement and the effectiveness of digital health interventions in older people living with neurocognitive disorders.

**International Registered Report Identifier (IRRID):**

PRR1-10.2196/65515

## Introduction

People aged 65 years and older represent 19% of the Canadian population and are expected to reach 25% by 2030 [1]. With the accelerated aging of the population, the prevalence of both major and mild cognitive disorders is increasing [2]. Cognitive disorders correspond to an alteration in one or more cognitive functions, regardless of the mechanism involved, its origin, or reversibility, and may be neurological, psychiatric, drug induced, and so on [3]. The prevalence of major cognitive disorders, such as Alzheimer disease, doubles every 5 years after the age of 65 years [4]. In 2015-2016, a total of 87% of residents in Canadian nursing homes had some type of cognitive impairment, 69% had dementia, 50% had behavioral disorders, and 31% had depression [5]. Of these residents, 82% required assistance with their care or were dependent on all care related to activities of daily living [5]. At the same time, the proportion of older people with dementia living outside a long-term care facility or nursing home was 61%, including 69% of those aged younger than 80 years and 58% of those aged 80 years and older [6]. The consequences of these cognitive disorders, which manifest themselves as a decline in performance in one or more cognitive domains [7], are often far-reaching for the individual, his or her family, health care professionals, and society. In Canada, the economic burden incurred by the health care system for caring for people with cognitive impairment was around CAD $10.4 billion (equivalent to US $7.2 billion) in 2016 and would reach CAD $16.6 billion (equivalent to US$ 11.5 billion) by 2031 [8].

Such a picture calls for mechanisms to maintain cognitive health in older adults. There are 2 main ones: vascular health and cognitive reserve [9]. Some of the protective factors acting on these mechanisms have been identified. First, physical activity contributes to maintaining vascular health and developing cognitive reserve, making it a preferred intervention for preventing cognitive impairment or delaying its progression [10]. Second, social participation, either direct or indirect contact between individuals in which information, attitudes, and norms are shared, has been shown to be a protective factor against cognitive decline [11].

Finally, cognitive engagement is associated with an increase in cognitive reserve [12]. This factor has 2 components: cognitive stimulation and cognitive training. On the one hand, cognitive stimulation is achieved through individual or group activities that involve different cognitive functions and consist of new learning activities, such as learning a new language [12]. Cognitive training, on the other hand, is based on the repetition of standardized tasks that target specific cognitive functions, such as the use of sophisticated video games [12]. These key protective factors (eg, physical activity, social participation, and cognitive engagement) can be promoted in a variety of ways, notably through the use of digital health technologies.

According to Health Canada, digital health technologies include stand-alone software applications as well as integrated software systems and instruments that can be used with platforms such as computers, smartphones, tablets, wearable devices, and environmental sensors [13,14]. Digital technologies for older adults, also known as gerontechnologies, have been used in many aspects of health care for older adults [2]. For example, mobile apps used to stimulate cognitive functions, connected devices to monitor physical activity, and digital platforms to facilitate communication with loved ones or consultation with health care professionals can be found among older people living with neurocognitive disorders [15]. Several studies have demonstrated the positive impact of digital health technologies on various aspects of an older person’s life, such as health, housing, services and transactions, mobility and transportation, access to information, communication and work, leisure, and personal fulfillment [16]. These technologies can improve access to health information, enable faster diagnosis and treatment, and improve access to care and services for older people with cognitive impairment at home, in health care facilities, and in rural and remote communities [13,17].

However, the potential benefits associated with the use of these technologies cannot be realized without the engagement of the people who use them. Thus, despite the growing interest in digital health technologies and the availability of evidence on the feasibility and effectiveness of health interventions using these technologies in a cognitively impaired population [18], little is known about the engagement of older people with cognitive impairment with these tools. Nevertheless, the success of digital health interventions depends on their adoption and optimal use, which implies an experienced and ongoing engagement of the people who use them. For example, some people may have access to the technology and possess the skills to use it but choose not to engage with a digital health service [19]. Other people engage with these technologies and then decide to abandon them. A systematic review and meta-analysis of attrition and adherence in smartphone-based interventions for mental health problems found attrition rates as high as 30% and very low adherence rates ranging from 2% to 10% [20]. Several factors could influence attrition in digital health interventions. In their study of barriers to adoption of mobile phone–based mental health interventions by older adults, Pywell et al [21] highlighted factors that may lead older adults to disengage. For example, participants felt that if they did not make sufficient progress while using digital health technologies, if they did not gain a better understanding of their symptoms and how they might affect them, or if they had to invest a lot of time and effort, then they would give up. These findings underscore the importance of increasing engagement, as there is evidence that higher levels of engagement are associated with better cognitive health [20].

For older people living with cognitive impairment, engagement with digital health technologies can be particularly challenging, and even more so for people from cultural or linguistic minority groups. Indeed, digital health technologies may contribute to increasing health inequalities due to systematic differences and people’s ability to engage with digital platforms [19].

The engagement of older people with cognitive impairment with digital health technologies is therefore a key factor in maximizing the benefits of these tools. This engagement can be influenced by conditions related to the design of digital health technologies, data security and confidentiality, and support for older people with cognitive impairment. However, other factors may also influence the level of engagement with digital health technologies in this population. Indeed, it is essential to understand the concept of engagement, how it is assessed, and how it relates to the effectiveness of digital health interventions for the prevention and management of cognitive disorders in older people. This understanding can inform the development and implementation of digital health interventions that are better adapted to this population, thus contributing to their health and well-being.

The purpose of this scoping review is to describe how the engagement of older adults with cognitive impairment with digital health technologies is conceptualized and assessed, and how this engagement relates to the effectiveness of digital health interventions.

Our key synthesis questions are as follows: (1) How is engagement conceptualized and operationalized in the context of studies of digital health technologies for older adults with cognitive impairment? (2) What measures are used to assess the level of engagement of older adults with cognitive impairment with digital health technologies? (3) What facilitates or limits the engagement of older adults with cognitive impairment with digital health technologies? and (4) What are the relationships between the level of engagement and the effectiveness of digital health interventions for older adults with cognitive impairment?

## Methods

### Overview

We will use the scoping review method based on the framework of Arksey and O’Malley [22], as improved by Levac et al [23]. In addition, we will use a systematic approach to conduct the scoping review, following the Joanna Briggs Institute recommendations for this type of review [24]. We will also follow the PRISMA-ScR (Preferred Reporting Items for Systematic Reviews Extension for Scoping Reviews) [25] to guide the scoping review approach and ensure reproducibility.

### Eligibility Criteria

Inclusion and exclusion criteria are listed in Textbox 1.

**Inclusion and exclusion criteria.**

**Inclusion criteria**

- We will include all types of evidence that meet the PCC (Population, Concept, Context) criteria.
- Participants or population: we will include all studies of people aged 65 years and older with cognitive impairment and their family caregivers.
- Concept: we will consider engagement with digital health technologies as a central concept in this scoping review.
- Context: we will include studies that address engagement with digital health technologies in all types of contexts (care setting, retirement home, community, etc) without geographic limitations.

**Exclusion criteria**

- We will exclude studies that address the concepts of use, adherence, compliance, participation, and adoption, which are often used interchangeably with the concept of engagement in studies.

### Search Strategy

The search strategy (Multimedia Appendix 1) was developed in collaboration with a librarian experienced in systematic reviews (FB). The research team conducted an iterative review, and all relevant comments on the research strategy were incorporated into the final version. The final version was approved by all members of the research team. A specific search strategy (Multimedia Appendix 1) combining concepts related to engagement and digital health was formulated for each of the following databases: MEDLINE (Ovid), Embase, CINAHL, and Web of Science, in addition to the Google Scholar search engine. The search strategy (Multimedia Appendix 1) was not restricted by time constraints. Gray literature, including government websites and documents, dissertations or theses, and conference abstracts, may be consulted as needed to supplement information from scholarly articles. The search will also include a manual search, and bibliographies of pertinent studies will be reviewed for additional relevant references.

### Data Collection

All search results in the various databases were exported to Covidence [26], a collaborative knowledge synthesis management software that automatically eliminates duplicates. This software allows double-blind evaluation at both stages of study selection. Independent and blind assessment of inclusion and exclusion criteria will be performed by at least 2 reviewers, first based on titles and abstracts, and second based on full texts. Any conflict will be resolved by consensus and finally by the principal investigator. For included studies at this stage, reviewers will read the full texts to further assessment of eligibility for the final inclusion. A PRISMA (Preferred Reporting Items for Systematic Reviews and Meta-Analyses) flowchart will be used to describe the identification of studies, the selection process, and the application of inclusion and exclusion criteria [27].

### Data Extraction

A preformatted data extraction grid will be developed by the research team. It will be used to compile the results extracted from the selected relevant studies. The extraction itself will be performed independently by two members of the research team and then validated by a senior member (MPG or MS). Disagreements will be resolved by consensus within the research team or, if necessary, by a third party. Data to be extracted include study characteristics (ie, title, year of publication, authors, and country where the study took place), intervention (type of engagement conceptualization and type of engagement measurement tools), context (care setting and community characteristics), participants (target population, stage of cognitive impairment, number of participants, and sample size), methods (study design, inclusion and exclusion criteria, and methodological quality), and outcome measured (qualitative and quantitative). We will highlight specific outcomes related to sex, gender, and other identity characteristics documented in the selected studies in line with the PROGRESS-Plus (place of residence; race, ethnicity, culture, and language; occupation; gender and sex; religion; education; socioeconomic status; social capital–personal characteristics associated with discrimination; features of relationships; and time-dependent relationships) health equity framework [19].

### Data Summary

We will conduct descriptive analyses using concept mapping to provide an overview of the information contained in the selected documents. We will first perform a narrative synthesis, grouping the findings by topic according to the initial research questions of the scoping review. We will then produce graphical representations of the results, depending on the nature of the information analyzed.

## Results

The development of the search strategy and the completion of the selection phases of the review were completed in July 2024. Data extraction and analysis began in August 2024, and results are expected to be available in November 2024.

## Discussion

### Overview

The results of this scoping review should provide evidence on how engagement with digital health technologies by older people with cognitive impairment is conceptualized, measured, and related to the effectiveness of digital behavior change interventions.

### Main Contributions of This Scoping Review

Several literature reviews have explored the concept of engagement [28-31]. Kelders et al [29] showed the variety of areas in which engagement has been studied. These include areas such as work, society, commerce, digital, and health. On the one hand, some authors have focused on conceptualizing engagement with digital behavior change interventions and tools for measuring engagement [30], while others have focused on patient engagement with digital health [31]. On the other hand, it should be noted that one review examined digital engagement among older people but focused on barriers and facilitators and was not specifically targeted at older people with cognitive impairment [16]. Another review examined the effectiveness of digital health solutions among older people with cognitive impairment [15], without focusing on their engagement with digital health technologies. Thus, to our knowledge, our review is the first attempt to examine the engagement of older people with cognitive impairment with digital health technologies. Beyond descriptive data, the synthesis of evidence by study type, intervention, context, methods, and outcomes will provide beacons to support the development of innovative interventions that promote the engagement of older people living with cognitive impairment with digital health technologies. In addition, our review will provide guidance for the evaluation of the effectiveness of these interventions through an analysis of tools for measuring the engagement of older people living with cognitive impairment toward digital health solutions.

### Potential Benefits and Next Steps

Engaging patients in the responsible management of their health is widely recognized as a means of overcoming challenges in the health care sector [28]. For this reason, it is generally accepted that some level of engagement is necessary for interventions to be effective [30]. However, it should be noted that patient engagement in the development, adoption, use, and evaluation of digital technologies in health care is not as advanced as in the traditional area of patient engagement in health care service delivery [31]. In the field of digital health, particularly in the case of older people with cognitive impairment, there is a lack of clarity in how engagement with digital technologies is conceptualized [32]. The results of this scoping review will provide a better understanding of the conceptualization and operationalization of engagement with digital technologies in health care by focusing on the experiences of older people living with cognitive impairment. The approach will promote the production of rigorous results while being adapted to the needs of older people with cognitive impairment, their caregivers, or the groups or institutions using this knowledge. The results of this scoping review will identify priority needs for knowledge users that can serve as a basis for the development of strategies to promote the engagement of older people with cognitive impairment with digital health technologies. The knowledge gained will inform further research for the development and implementation of digital health technologies that are better adapted to older people with cognitive impairment, thereby fostering their engagement and achieving the expected benefits for their health and well-being.

### Conclusion

The results of this scoping review will provide a comprehensive mapping of the conceptualization and measurement of engagement of older people with digital health technologies. Such evidence will enable technology providers to develop digital technologies that focus on engagement, and policy makers and practitioners to develop engaging digital health interventions that promote cognitive health and prevent cognitive impairment in older people.

## Appendix

Multimedia Appendix 1 Search strategy.

Multimedia Appendix 2 PRISMA-P (Preferred Reporting Items for Systematic Review and Meta-Analysis Protocols) checklist.

Multimedia Appendix 3 Peer review report from the Brain Health and Reduction of Risk for Age-related Cognitive Impairment - Knowledge Synthesis and Mobilization Grants Committee (Canadian Institutes of Health Research, Canada).

## Abbreviations

PRISMA: Preferred Reporting Items for Systematic Reviews and Meta-Analyses
PRISMA-ScR: Preferred Reporting Items for Systematic Reviews and Meta-Analyses Extension for Scoping Reviews
PROGRESS-Plus: place of residence; race, ethnicity, culture, and language; occupation; gender and sex; religion; education; socioeconomic status; social capital–personal characteristics associated with discrimination; features of relationships; and time-dependent relationships

## Data Availability

Data on the search strategy for this review are available in Multimedia Appendix 1. All data generated or analyzed during this study (including the PRISMA [Preferred Reporting Items for Systematic Reviews and Meta-Analyses] flowchart) will be included as multimedia appendices. Databases created during the data extraction process will be made available upon reasonable request.
