# Supplementary material for: Engaging Older Adults With Cognitive Impairment in Digital Health Technologies: Protocol for a Scoping Review
Source: JMIR Res Protoc. 2025 Jun 3;14:e65515. doi: 10.2196/65515 (PMC12174866; doi:10.2196/65515)
Supplement: Multimedia Appendix 2 [file resprot_v14i1e65515_app2.docx]

**PRISMA-P (Preferred Reporting Items for Systematic review and Meta-Analysis Protocols) 2015 checklist: recommended items to address in a systematic review protocol***

| Section and topic | Item No | Checklist item |
| --- | --- | --- |
| ADMINISTRATIVE INFORMATION | | |
| Title: |  |  |
| Identification | 1a | **Identify the report as a protocol of a systematic review**  Engaging older adults with cognitive impairment in digital health technologies: A systematic scoping review protocol |
| Update | 1b | **If the protocol is for an update of a previous systematic review, identify as such**  This is not an update to the protocol. |
| Registration | 2 | **If registered, provide the name of the registry (such as PROSPERO) and registration number**  Our scoping review protocol was registered with the Open Science Framework (OSF) on February 23, 2024 and last updated on March 14, 2024 with the DOI https://doi.org/10.17605/OSF.IO/UKY3Q. |
| Authors: |  |  |
| Contact | 3a | **Provide name, institutional affiliation, e-mail address of all protocol authors; provide physical mailing address of corresponding author**  **Corresponding Author**: Marie-Pierre Gagnon; Email: [marie-pierre.gagnon@fsi.ulaval.ca](about:blank)  Author affiliations:  -1. Faculty of Nursing Sciences, Laval University, 1050 Medicine Avenue, Quebec (Quebec) G1V 0A6  -2. VITAM, Research Center for Sustainable Health, 2480 Canardière Road, Quebec (Quebec) G1J 2G1  **Co-authors**: [Sié Mathieu Aymar Romaric Da](about:blank) 1,2, MSc;  [Maxime Sasseville](about:blank)1,2, RN, PhD; [Marie-Soleil Hardy](about:blank)1,2, PhD; Idrissa Beogo 3, PhD; [Amédé Gogovor](about:blank) 4, PhD; [Samira Amil](about:blank) 2, RD; Achille R Yameogo1,2, MSc; Frédéric Bergeron 2, MSI; [Anik Giguère](about:blank) 4, PhD; [Annie LeBlanc](about:blank) 4, PhD;  James Plaisimond1,2, MSc; Carole Rivard-Lacroix (5).  Co-authors affiliations:  -1. Faculty of Nursing Sciences, Laval University, 1050 Medicine Avenue, Quebec (Quebec) G1V 0A6  -2. VITAM, Research Center for Sustainable Health, 2480 Canardière Road, Quebec (Quebec) G1J 2G1  -3. School of Nursing, Ottawa University, 451 Smyth Rd, Ottawa, ON K1H 8M5  -4. Faculty of Medicine, Laval University, 1050 Medicine Avenue / Quebec, QC CAN G1V 0A6  -5. Institute for Aging and Social Participation of Seniors, Laval University. |
| Contributions | 3b | **Describe contributions of protocol authors and identify the guarantor of the review**  Funding acquisition, supervision, M-PG; conception, SMAR.D, M.S, M-PG; methodology, SMAR.D, F.B, M-PG; drafting of protocol, SMAR.D; final drafting and editing of protocol, all authors. M-PG is the guarantor of the review. |
| Amendments | 4 | **If the protocol represents an amendment of a previously completed or published protocol, identify as such and list changes; otherwise, state plan for documenting important protocol amendments**  The protocol is not a modification of a previously completed or published protocol. |
| Support: |  |  |
| Sources | 5a | **Indicate sources of financial or other support for the review**  This project was funded by the Canadian Institutes of Health Research (Operating Grant: Knowledge Synthesis and Mobilization, Brain Health, and Reducing the Risk of Age-Related Cognitive Impairment). |
| Sponsor | 5b | **Provide name for the review funder and/or sponsor**  Canadian Institutes of Health Research. |
| Role of sponsor or funder | 5c | **Describe roles of funder(s), sponsor(s), and/or institution(s), if any, in developing the protocol**  The Canadian Institutes of Health Research has no influence over the research process or the publication of results. |
| INTRODUCTION | | |
| Rationale | 6 | **Describe the rationale for the review in the context of what is already known**  [Title: Engaging older adults with cognitive impairment in digital health technologies: A systematic scoping review protocol]  People aged 65 and over make up 19% of the Canadian population and are expected to reach 25% by 2030. As the population ages, the prevalence of mild and severe cognitive impairment is increasing. The consequences of these cognitive disorders, which manifest as a decline in performance in one or more cognitive domains, are often serious for the individual, their family, health care professionals and society. In Canada, the economic burden on the health care system of caring for people with cognitive disorders was approximately $10.4 billion in 2016 and is expected to increase to $16 billion by 2030. The use of digital health technologies is an opportunity to reduce the burden on health care systems and improve the cognitive health of older people.  Several studies have demonstrated the positive impact of digital health technologies on various aspects of an older person's life, such as health, housing, services and transactions, mobility and transportation, access to information, communication and work, leisure and personal fulfillment. These technologies can improve access to health information, enable faster diagnosis and treatment, and improve access to care and services for cognitively impaired older people at home, in healthcare facilities, and in rural and remote communities.  However, the potential benefits associated with the use of these technologies cannot be realized without the engagement of the people who use them. Several factors influence older people's engagement with digital health technologies. In their study of barriers to adoption of mobile phone-based mental health interventions by older people, Pywell et al (2020) highlighted factors that could lead older people to disengage. For example, participants felt that they would give up if they did not make sufficient progress while using digital health technologies, if they did not gain a better understanding of their symptoms and how they might affect them, or if they had to invest a lot of time and effort. A systematic review and meta-analysis of attrition and adherence in smartphone-based interventions for mental health problems found attrition rates of up to 30% and very low adherence rates of between 2 and 10%.  These findings suggest the importance of increasing older adults' engagement with digital health technologies, as there is evidence that high levels of engagement are associated with better cognitive health. For this reason, some studies have focused on the concept of engagement in older adults, but to our knowledge, none have focused on the engagement of older adults with cognitive impairment with digital health technologies. Therefore, it is important to understand the concept of engagement, how it is assessed, and how it relates to the effectiveness of digital health interventions for the prevention and management of cognitive impairment in older adults with cognitive impairment. |
| Objectives | 7 | **Provide an explicit statement of the question(s) the review will address with reference to participants, interventions, comparators, and outcomes (PICO).**  As this is a scoping review, the eligibility criteria follow the Population, Concept and Context (PCC) approach rather than the PICO.  The purpose of this systematic scoping review is to describe how the engagement of cognitively impaired older adults with digital health technologies is conceptualized and assessed, and how this engagement relates to the effectiveness of digital health interventions. To this end, the proposed scoping review will answer the following questions:  1. How is engagement conceptualized and operationalized in studies of digital health technologies for older adults with cognitive impairment?  2. What measures are used to assess the level of engagement of cognitively impaired older adults with digital health technologies?  3. What facilitates or limits cognitively impaired older adults' engagement with digital health technologies?  4. What are the relationships between the level of engagement and the effectiveness of digital health interventions for cognitively impaired older adults? |
| METHODS | | |
| Eligibility criteria | 8 | **Specify the study characteristics (such as PICO, study design, setting, time frame) and report characteristics (such as years considered, language, publication status) to be used as criteria for eligibility for the review**  **Overview**  This knowledge synthesis will be conducted using the scoping review method and will be based on the framework of Arksey and O'Malley [20], as improved by Levac et al [21]. In addition, we will use a systematic approach to conduct the scoping review, following the recommendations of the Joanna Briggs Institute (JBI) for this type of review [22]. We will also follow the Preferred Reporting Items for Systematic Reviews extension for Scoping Reviews (PRISMA-ScR) [23] to guide the scoping review approach and ensure reproducibility. The search strategy was not limited by time constraints.  **Inclusion criteria:**  Includes all types of evidence that meet the PCC (population, concept, context) criteria.  Participants or population: We will include all studies of cognitively impaired people aged 65 years and older and their family caregivers.  Concept: We will consider engagement with digital health technologies as a central concept in this scoping review.  Context: We will include studies that address engagement with digital health technologies in all types of contexts (care setting, retirement home, community, etc.) without geographic limitations.  **Exclusion criteria:**  Studies that address the concepts of use, adherence, compliance, participation, and adoption, which are often used interchangeably with the concept of engagement in studies, were excluded. |
| Information sources | 9 | **Describe all intended information sources (such as electronic databases, contact with study authors, trial registers or other grey literature sources) with planned dates of coverage.**  A specific search strategy combining concepts related to engagement and digital health was formulated for each of the following databases: Medline (Ovid), Embase (Embase.com), CINAHL, Web of Science, and the Google Scholar search engine. Gray literature, including government websites and documents, theses and dissertations, and conference abstracts, may be consulted as needed to supplement information from scholarly articles. The search will also include a manual search, and bibliographies of relevant studies will be reviewed for additional relevant references. |
| Search strategy | 10 | **Present draft of search strategy to be used for at least one electronic database, including planned limits, such that it could be repeated**  The search strategy was developed in collaboration with a librarian experienced in systematic reviews (FB). The research team conducted an iterative review and all relevant comments on the search strategy were incorporated into the final version. The final version was approved by all members of the research team. A specific search strategy combining terms related to engagement and digital health was formulated for each of the following databases: Medline (Ovid), Embase (Embase.com), CINAHL, Web of Science, in addition to the Google Scholar search engine. Gray literature, including government websites and documents, theses and dissertations, and conference abstracts, may be consulted as needed to supplement information from scholarly articles. The search will also include a manual search, and bibliographies of relevant studies will be reviewed for additional relevant references.  **Draft MEDLINE search - Ovid interface**  Database limit: no database limit has been applied.   1. Patient Participation/ OR (Engage* OR Involv* OR Participation OR disengag*).ti,ab,kf OR (microengagement OR macroengagement).ti,ab,kf 2. Digital Technology/ OR Digital Health/ OR Smartphone/ OR Computers, Handheld/ OR Mobile Applications/ OR (Digital adj1 (Technolog* OR Health)).ti,ab,kf OR (mobile adj2 (application? OR technolog* OR app OR device? OR apps OR Phone)).ti,ab,kf OR Smartphone?.ti,ab,kf OR (tablet adj1 (application? OR computer?)).ti,ab,kf OR "chat bot?".ti,ab,kf OR chatterbot?.ti,ab,kf OR chatbot?.ti,ab,kf OR medbot?.ti,ab,kf OR "chatter bot?".ti,ab,kf OR smart bot?.ti,ab,kf OR smartbot?.ti,ab,kf OR (Conversational adj2 (assistant OR interface OR agent? OR system OR computer OR bot? OR AI)).ti,ab,kf OR ((virtual OR intelligent OR computer OR AI OR "artificial intelligence" OR embodied) adj2 agent?).ti,ab,kf 3. exp Aged/ OR Aged.ti,ab,kf OR geriatric?.ti,ab,kf OR elder*.ti,ab,kf OR senior?.ti,ab,kf OR ((old OR oldest? OR older?) adj2 (person? OR adult? OR individual? OR people OR patient?)).ti,ab,kf 4. Mental Disorders/ OR exp Neurocognitive Disorders/ OR Memory Disorders/ OR Schizophrenia/ OR exp Parkinsonian Disorders/OR ((Mental OR Neurocogniti* OR Psych* OR Cogniti*) adj2 (Disorder? OR Illness* OR Disease? OR Dysfunction OR Decline OR Deterioration OR Impairment?)).ti,ab,kf OR (Memory adj2 (Disorder? OR Deficit OR Loss* OR Impairment?)).ti,ab,kf OR Alzheimer?.ti,ab,kf OR Schizophrenia?.ti,ab,kf OR Parkinson?.ti,ab,kf OR Huntington?.ti,ab,kf OR Delirium.ti,ab,kf OR dementia?.ti,ab,kf OR Amnesia?.ti,ab,kf OR (Lewy adj1 (Body OR Bodies)).ti,ab,kf |
| Study records: |  |  |
| Data management | 11a | **Describe the mechanism(s) that will be used to manage records and data throughout the review**  All search results in the various databases were exported to the online collaboration tool Covidence (Veritas Health Innovation), a collaborative knowledge synthesis management software that automatically eliminates duplicates. This software allows for double-blind evaluation at both stages of study selection. |
| Selection process | 11b | **State the process that will be used for selecting studies (such as two independent reviewers) through each phase of the review (that is, screening, eligibility and inclusion in meta-analysis)**  Independent and blind assessment of inclusion and exclusion criteria will be performed by at least 2 reviewers, firstly based on titles and abstracts and secondly based on full texts. Conflicts will be resolved by consensus and ultimately by the principal investigator. For studies included at this stage, reviewers will read the full texts to further assess eligibility for final inclusion. A PRISMA flowchart will be used to describe the identification of studies, the selection process, and the application of inclusion and exclusion criteria. |
| Data collection process | 11c | **Describe planned method of extracting data from reports (such as piloting forms, done independently, in duplicate), any processes for obtaining and confirming data from investigators**  A data extraction grid will be developed by the research team. It will be used to compile the results extracted from the selected relevant studies. The extraction itself will be performed by two members of the research team and then validated by a senior member (MPG or MS). Disagreements will be resolved by consensus within the research team or, if necessary, by a third party. |
| Data items | 12 | **List and define all variables for which data will be sought (such as PICO items, funding sources), any pre-planned data assumptions and simplifications**  Data to be extracted include study characteristics (i.e., title, year of publication, authors, country in which the study was conducted), intervention (type of engagement conceptualization, type of engagement measurement tools), context (care setting, community characteristics), participants (target population, stage of cognitive impairment, number of participants, sample size), methods (study design, inclusion and exclusion criteria, methodological quality), and outcomes measured (qualitative and quantitative). We will highlight specific outcomes related to sex, gender and other identity characteristics documented in the selected studies in line with the PROGRESS-Plus health equity framework. |
| Outcomes and prioritization | 13 | **List and define all outcomes for which data will be sought, including prioritization of main and additional outcomes, with rationale**  We will conduct descriptive analyses using concept mapping software (Xmind) to provide an overview of the information contained in the selected documents. We will first conduct a narrative synthesis, grouping the findings by topic according to the initial research questions of the scoping review. We will then produce graphical representations of the findings, depending on the nature of the information analyzed.  The development of the search strategy and completion of the selection phase of the review will be completed in July 2024. Data extraction and analysis will begin in August 2024, and results are expected to be available in October 2024. |
| Risk of bias in individual studies | 14 | **Describe anticipated methods for assessing risk of bias of individual studies, including whether this will be done at the outcome or study level, or both; state how this information will be used in data synthesis**  Not applicable as this is a scoping review. |
| Data synthesis | 15a | **Describe criteria under which study data will be quantitatively synthesised**  Not applicable as this is a scoping review. |
|  | 15b | **If data are appropriate for quantitative synthesis, describe planned summary measures, methods of handling data and methods of combining data from studies, including any planned exploration of consistency (such as I^2^, Kendall’s τ)**  Not applicable as this is a scoping review. |
|  | 15c | **Describe any proposed additional analyses (such as sensitivity or subgroup analyses, meta-regression)**  Not applicable as this is a scoping review**.** |
|  | 15d | **If quantitative synthesis is not appropriate, describe the type of summary planned**  Not applicable as this is a scoping review. |
| Meta-bias(es) | 16 | **Specify any planned assessment of meta-bias(es) (such as publication bias across studies, selective reporting within studies)**  Not applicable as this is a scoping review. |
| Confidence in cumulative evidence | 17 | **Describe how the strength of the body of evidence will be assessed (such as GRADE)**  Not applicable as this is a scoping review. |

*** It is strongly recommended that this checklist be read in conjunction with the PRISMA-P Explanation and Elaboration (cite when available) for important clarification on the items. Amendments to a review protocol should be tracked and dated. The copyright for PRISMA-P (including checklist) is held by the PRISMA-P Group and is distributed under a Creative Commons Attribution Licence 4.0.**

*From: Shamseer L, Moher D, Clarke M, Ghersi D, Liberati A, Petticrew M, Shekelle P, Stewart L, PRISMA-P Group. Preferred reporting items for systematic review and meta-analysis protocols (PRISMA-P) 2015: elaboration and explanation. BMJ. 2015 Jan 2;349(jan02 1):g7647.*
