# Supplementary material for: Engaging Older Adults With Cognitive Impairment in Digital Health Technologies: Protocol for a Scoping Review
Source: JMIR Res Protoc. 2025 Jun 3;14:e65515. doi: 10.2196/65515 (PMC12174866; doi:10.2196/65515)
Supplement: Multimedia Appendix 3 [file resprot_v14i1e65515_app3.pdf]

| | |
|---|---|
| **Review Type/Type d'évaluation:** | SO Notes /Notes de l'agent scientifique |
| **Name of Applicant/Nom du chercheur:** | Gagnon, Marie-Pierre |
| **Application No./Numéro de demande:** | 503555 |
| **Agency/Agence:** | CIHR/IRSC |
| **Competition/Concours:** | 2023-06-22 Operating Grant: Brain Health and Reduction of Risk for Age-related Cognitive Impairment - Knowledge Synthesis and Mobilization Grants/Subvention de fonct. : Synthèse et mobilisation des connaissances : santé cérébrale et réduction du risque de déficience cognitive liée à l'âge |
| **Committee/Comité:** | BHCIA : Knowledge Synthesis and Mobilization Grants/Synthèse et mobilisation des connaissances : SCDCV |
| **Title/Titre:** | Engagement des personnes âgées envers les technologies de santé numérique : Un examen de la portée systématique |

**Assessment/Évaluation:**

The committee felt that this application was innovative and had the potential to create meaningful and valuable results for the field of health technology. The proposal is well-written. Technology users and other knowledge users and their carers are included, noted as a plus. The team is multidisciplinary with a solid track record. The committee noted that this was a particularly important, timely, and relevant topic.

Concerns included that the team may not be able to generate enough data points for their scoping review and thus affect their ability to meet the knowledge synthesis objective. A related concern was the time limits on the literature search that might exclude the technological experience of older stakeholders. More importantly, using a scoping review versus more in-depth knowledge synthesis to lead to recommendations was a major concern.

The value of any information arising from subgroup analyses was also a concern given the potential of small sample size. This issue may also impact the feasibility of conducting analyses related to sex and gender.

The knowledge mobilization plan was adequate but not particularly innovative. A more robust description of the plan and expected impact of the workshops would have been useful to the committee. Including computer scientist and/or software developer expertise on the team was discussed as something to be considered. More information on the qualitative component and the value of expanding the analyses to include mixed methods, rather than the strong quantitative focus, in order to provide richer and deeper findings was raised.

No concerns regarding the budget were raised. The committee noted that ethics approval would be required given that information will be gathered from stakeholders and participants.
